# Examining potential effects of non-occupational post-exposure prophylaxis (nPEP) on sexual behaviors of Chinese men who have sex with men: a cross-sectional study

**DOI:** 10.1186/s12889-021-10283-0

**Published:** 2021-01-26

**Authors:** Haochu Li, Ran Wei, Jason J. Ong, Eunsook Kim, Traci L. Weinstein, Xiaofu Ning, Wei Ma

**Affiliations:** 1 Department of Epidemiology, School of Public Health, Cheeloo College of Medicine, Shandong University, 44 West Wenhua Road, Jinan, 250012 Shandong Province China; 2 Monash University Central Clinical School, Melbourne, VIC 3004 Australia; 3 Department of Educational and Psychological Studies, University of South Florida, Tampa, FL 33620-9951 USA; 4 Department of Psychology, Rhode Island College, Providence, RI 02908-1924 USA; 5 Peking University School of Pharmaceutical Sciences, Beijing, 100191 China

**Keywords:** Anticipated behavioral change, Condom use, number of sex partners, Non-occupational post-exposure prophylaxis (nPEP), Men who have sex with men, China

## Abstract

**Background:**

In China, non-occupational post-exposure prophylaxis (nPEP) is not a conventional service yet and nPEP related studies are very few. Recently, China’s 13th Five Year Action Plan for HIV/AIDS Containment and Prevention examines the feasibility of including nPEP as one of the national strategies for HIV prevention. However, there is a concern that nPEP use might exacerbate high-risk sexual activities. In order to facilitate a research-based policy making of routinizing nPEP services, the current study examined potential effects of nPEP use on condom use and number of sexual partners among Chinese men who have sex with men (MSM) .

**Methods:**

A cross-sectional survey was conducted in two cities of China in November and December 2018. Descriptive analyses of participants’ sociodemographic and behavioral characteristics were conducted using SPSS 24.0. Mplus 7.4 was used to conduct confirmatory factor analysis and structural equation modeling.

**Results:**

The sample included 419 Chinese MSM with a mean age of 28.04 (SD = 9.71). Participants reported more positive anticipation of their own behaviors than other MSM’s behaviors regarding condom use and number of sexual partners if nPEP were to be routinized in China. About 60% of participants reported discrepancies between anticipated individual and population behaviors as a potential result of routinization of nPEP services. Anticipated individual behavioral change was positively related to age and duration of residence in the current city, and negatively related to education. Anticipated population behavioral change was positively related to age. Anticipated behavioral discrepancy was positively related to being ethnic minority and never married.

**Conclusions:**

These findings identify a high-risk subgroup of MSM, who reported they would use condoms less and/or have more sexual partners when nPEP becomes available. This subgroup of MSM might benefit from targeted health interventions. Moreover, there is a clear discrepancy between anticipated individual and population behavioral changes regarding future routinization of nPEP services, suggesting incorporating nPEP services as a means of community development for MSM.

## Background

Non-occupational post-exposure prophylaxis (nPEP) is effective in reducing human immunodeficiency virus (HIV) transmission and involves the administration of antiretroviral drugs within 72 h of an exposure where there is a substantial likelihood for HIV transmission and continuing the treatment for a total of 28 days [1]. nPEP is recommended when a person’s sexual partner is HIV-positive with a detectable or unknown viral load or there was condomless anal sex with men who have sex with men (MSM) of unknown HIV status [1]. nPEP programs have been implemented in many resource-rich countries to complement other effective HIV prevention strategies [2]. The World Health Organization has also provided guidance on the use of nPEP [3]. Studies reported that the use of antiretrovirals after a high risk exposure can reduce the likelihood of HIV infection by 79–81% [4] and nPEP is cost-effective when targeted to high-risk populations [5, 6]. However, there is a perception that nPEP might exacerbate high-risk sexual activities, also called sexual risk behavior disinhibition [7, 8]. In this regard, studies to estimate potential nPEP effects on sexual behaviors among key populations, such as MSM, are warranted.

The current literature show that the relationship between nPEP and sexual risk behavior is not conclusive. Some studies confirm that disinhibition of sexual risk behavior is a concern for individuals taking nPEP. A study from the United States (US) found a significant proportion (21%) of MSM reported condomless sex and 11% of MSM reported condomless sex with HIV-positive or HIV status unknown partners during nPEP use [9]. A longitudinal study in the Netherlands found that the majority of the HIV seroconverters reported ongoing risk behavior after nPEP prescription [10]. Another longitudinal study in Australia reported an association between nPEP use and high risk of subsequent HIV infection among MSM [11]. A retrospective study in Australia also confirmed that nPEP users had a higher number of male partners, less consistent condom use, and injecting drug use history [12]. However, there are other studies that reported different results. A study in San Francisco reported that knowledge about the availability of nPEP did not lead to an increase in condomless anal sex among gay men [13]. Another longitudinal study also reported that most MSM (73%) had a decrease in high-risk sexual behaviors and most of them (85%) had no change in incident sexually transmitted infections (STI), indicating a lack of behavioral disinhibition [14]. Another randomized HIV-prevention trial among MSM in the US observed that, although nPEP users were a riskier group at baseline, previous nPEP use was not associated with higher odds of high risk sex at follow-up [15]. In Brazil, a cohort study also reported that nPEP use was not associated with increases in reported high-risk behavior [4]. These discrepancies require further exploration, which is a significant contribution of the current study.

Recently, China’s 13th Five-Year Action Plan for HIV/AIDS Containment and Prevention examined the feasibility of including nPEP as one of the strategies for HIV prevention. Yet, in China, there are few nPEP studies to date. One study published in a Chinese journal explored factors related to the need of nPEP and reported only 22.1% of MSM had heard about nPEP [16]. Another study described the introduction of nPEP services in an acquired immune deficiency syndrome (AIDS) designated hospital in Beijing, but did not report any impact of nPEP use on sexual risk behaviors [17]. In the English literature, nPEP studies from China are largely absent. This might be because nPEP is not a conventional service widely available in China yet, although some AIDS-designated hospitals provide nPEP on a case-by-case basis. Before nPEP is routinized nationwide, it is necessary to examine the potential for nPEP use to change sexually risky behaviors. The current study aims to explore the potential effects of nPEP use on individual and population sexual behaviors from the perspective of MSM if nPEP is routinized nationally.

The current study is guided by social cognitive theory, which conceptualizes an interactional causal structure as triadic reciprocal causation of behavior that involves a dynamic interplay among personal determinants, behavior and environmental influences [18]. Therefore, we modelled the relationships among personal determinants (e.g., age, education, HIV knowledge, nPEP knowledge), behavior (e.g., using nPEP, looking for male sexual partners through the Internet), and environmental factors (e.g., city of residence, duration of local residence in the current city) in the current study. We hypothesize that nPEP use and related knowledge might be related to MSM’s sexual risk behavior. Moreover, previous studies reported that nPEP use was associated with inconsistent condom use [12] and more sexual partners among MSM [19], supporting our hypothesis that MSM’s behaviors regarding condom use and number of sexual partners might change if nPEP is widely available. A study has reported a significant discrepancy between people’s actual risk behavior and their perception [20]. However, there are very few HIV studies examining anticipated behavioral discrepancy between individual and population. We adopted this concept from studies that examined perceived/anticipated behavioral or cognitive/emotion discrepancy in other health-related models [21, 22]. We hypothesize that nPEP use might influence nPEP knowledge, and it might influence anticipated individual and population behavioral changes and discrepancy directly and indirectly. People generally first learn about HIV, and then nPEP [23], and HIV and nPEP knowledge were associated with an intention to use nPEP [24]. In this regard, we hypothesize that HIV knowledge might have an effect on nPEP knowledge, and it might influence anticipated individual and population behavioral changes and discrepancy directly and indirectly.

## Methods

A cross-sectional study that explored Chinese MSM’s awareness, knowledge, and perceived need of nPEP, willingness to use nPEP, and sociodemographic factors was conducted in November and December, 2018. Two big cities in China, Shijiazhuang in the north and Xiamen in the south, were selected given that MSM populations are large and accessible in these two cities, and local centers for disease control and prevention and community-based organizations (CBOs) were able to help with the data collection. Local CBOs helped recruit participants. Inclusion criteria were: (1) biological males (assigned male sex at birth) aged 18 years and over; (2) had sex with another male in the past 12 months; (3) self-reported negative or were unaware of their HIV status; and (4) provided written informed consent and participated voluntarily in the survey. Exclusion criteria were: (1) self-reported HIV positive; (2) not able to complete the survey due to health problems (e.g., mental illness); and (3) unable to provide written informed consent.

### Survey measures

The structured survey instrument was iteratively developed by first referring to China’s HIV/AIDS sentinel surveillance questionnaire [25] and a scoping review of both domestic and foreign literature, revising the questionnaire according to the results of qualitative work, and consulting experts who have research or clinical experiences related to HIV and nPEP among MSM. The instrument covered topics such as socio-demographic information, HIV/AIDS knowledge, HIV-related behaviors (i.e., sexual behaviors, alcohol and drug use, and HIV testing), awareness of and need for nPEP, and potential impact of nPEP services on future sexual risk behaviors [26].

#### HIV knowledge

A scale with eight categorical items with three response options was used [25]. An example question is “Will infections of other sexually transmitted diseases increase the risk of getting HIV infection?” with answers of “Yes (1)”, “No (0)”, and “unknown (0)”. Responses were transformed into correct or incorrect binary items, treating unknown as incorrect in data analysis. Higher scores indicated better knowledge of HIV/AIDS. Latent variable modeling showed that the HIV Knowledge Scale’s reliability was 0.824 with 95% confidence interval (CI) of 0.796–0.853.

#### nPEP knowledge

An author-derived scale with nine items was used. An example question was “A person needs to make sure they are not infected with HIV before taking nPEP” with answers of “correct (1)”, “wrong (0)”, and “unknown (0)”. Higher scores indicated better knowledge of nPEP. Latent variable modeling showed that the nPEP Knowledge Scale’s reliability was 0.903 with 95% CI of 0.891–0.915.

#### HIV-related behaviors and utilization of services

Items assessing behaviors related to HIV risk and testing included number of instances of anal sex with males in the last week, number of male sexual partners in the last 6 months, condom use in the most recent anal sex with other males in the last 6 months, condom breakage, slippage or intentional removal when having sex with other males in the last 6 months, use of illicit drugs, frequency of drinking alcohol in the last 3 months, diagnosis of sexually transmitted disease (STD) in the past 12 months, frequency of HIV testing, and use of nPEP ever.

#### Socio-demographic information

Nine items assessed participants’ socio-demographic characteristics. Participants indicated their age, city of residence, duration of residence in the current city, education level, monthly income, marital status with women, sexual orientation, locations for looking for male sexual partners, and status of being a student.

#### Anticipated individual behavioral changes

Two author-derived categorical items were used, asking for participants’ opinions about future condom use behavior and number of sex partners regarding themselves. An example question is “If nPEP is promoted nationally, what change regarding your personal behavior of condom use will you have?” The answer was given as either decrease (1), no change (2), or increase (3). A higher score indicated higher positive impact of nPEP services on future risky behaviors.

#### Anticipated population behavioral changes

Two author-derived categorical items were used, asking for participants’ opinions about future condom use behavior and number of sex partners regarding the whole MSM population. An example question is “If nPEP is promoted nationally, what change regarding MSM population’s behavior of condom use will there be?” The answer was either given as decrease (1), no change (2), or increase (3). A higher score indicated higher positive impact of nPEP services on MSM population future risky behaviors.

#### Anticipated behavioral discrepancy

This variable was adopted and adjusted from other studies [20–22]. This was calculated as the sum of absolute values of anticipated individual change minus anticipated population change in condom use and the number of sexual partners respectively. Higher scores indicated larger discrepancy between anticipated individual and population behavior in condom use and number of sexual partners.

### Survey procedures

Before conducting the survey, investigators were trained to ensure they were familiar with the survey process and understood the content of the questionnaire. A convenience sampling method was used. Local CBOs invited potential participants when they came for HIV testing and organized volunteers to invite participants from MSM venues (e.g., gay saunas). After participants provided written informed consent, they received a questionnaire link from Wenjuanxing (a professional electronic survey platform) and personal keys, which ensured better authenticity of survey results. Participants completed the survey independently on their smart phone or computer. After the submission of the questionnaire, an audit was conducted by the research team, after which participants were provided 30 Yuan, equal to United States dollar (USD) 4.50, through Wechat Red Pack as compensation. A total of 423 questionnaires were received, and 419 of them were retained after four questionnaires were deleted due to a large number of questions unanswered.

### Statistical analysis

Software package for statistical analysis (SPSS) 24.0 was used to conduct a descriptive analysis of participants’ sociodemographic and behavioral characteristics. Mplus 7.4 was used to conduct confirmatory factor analysis (CFA) for measurement models and structural equation modeling (SEM) for estimation of research outcomes. Goodness-of-fit of the models was assessed using the comparative fit index (CFI), the Tucker-Lewis index (TLI), and the root mean square error of approximation (RMSEA). Acceptable model fit is determined by a RMSEA less than 0.08, and values of CFI and TLI greater than 0.90 [27, 28]. The CFA solutions were evaluated with acceptable overall goodness-of-fit, no focal areas of ill fit (absence of large modification indices and standardized residuals), and no out-of-range values in the parameter estimates [29]. Latent variable modeling was used to test the scale’s reliability [30]. Finally, SEM were conducted using anticipated behavioral changes and anticipated behavioral discrepancy as outcome variables respectively to estimate their relationships with nPEP knowledge, HIV knowledge, and sociodemographic and behavioral covariates (so called, a path model). We modelled nPEP knowledge as a mediator and tested the nPEP use and HIV knowledge effects on each outcome directly and indirectly through nPEP knowledge. In addition, the measurement models of nPEP knowledge, HIV knowledge, and each outcome variable were directly included in SEM (namely, structural regression model) to take into account measurement errors.

The structural regression model in SEM is a combination of measurement model (or CFA) and path model and allows researchers to model not only complex relations between variables such as mediation but also measurement errors explicitly [31–33]. Thus, researchers can conduct factor analysis and test research hypotheses simultaneously and obtain measurement-error-free estimates. For the estimation of CFA and SEM, we used weighted least squares with mean and variance adjusted (WLSMV) instead of commonly used maximum likelihood (ML) because the outcome variables in this study were categorical. Because multivariate normality cannot be assumed with categorical variables, the WLSMV is considered an appropriate estimator and the outperformance of WLSMV over ML was reported for categorical data when the number of categories was less than five [34–36].

## Results

### Sociodemographic and behavioral characteristics

The sample included 419 participants with a mean age of 28.04, with a standardized deviation (SD) of 9.71. The majority (78.8%) were aged between 18 and 34 years. Most (97.1%) were Han Chinese, and most (84.7%) lived in their current cities for more than 12 months. A majority (65.9%) had education of college or above, and a majority (67.8%) had monthly incomes of China Yuan (CYN) 5000 (about USD 743) or lower. Most (76.8%) never got married or cohabited with females. The majority (58.5%) did not have anal sex with males in the last week, while a significant proportion (38.7%) reported one to four episodes of anal sex and a small proportion (2.9%) were very sexually active (more than four times in the last week). Most (64.9%) drank alcohol in the last 3 months. Most participants (69.7%) reported testing for HIV in the last 12 months. Only 3.6% of participants reported use of nPEP ever. About 60% of participants reported discrepancies between anticipated individual and population behavioral change (see Table 1).

**Table 1:** Sociodemographic and behavioral characteristics of MSM in two cities of China, 2018 (*N* = 419)

	Number	Percent
Age (mean = 28.04, SD = 9.71, years)		
18–34	330	78.8
35–49	69	16.5
50–64	19	4.5
65+	1	0.2
City of residence		
North	217	51.8
South	202	48.2
Ethnicity		
Han	407	97.1
Non-Han (other ethnicity)	12	2.9
Duration of residence in the current city		
< 3 months	22	5.3
3–6 months	22	5.3
7–12 months	20	4.8
13–24 months	60	14.3
> 24 months	295	70.4
Education		
Illiterate	4	1.0
Primary school	5	1.2
Secondary school	47	11.2
High/Polytechnic school	87	20.8
College or above	276	65.9
Monthly income (CYN) ^a^		
< 1500	89	21.2
1500–3000	72	17.2
3001–5000	123	29.4
5001–8000	75	17.9
> 8000	49	11.7
Missing	11	2.6
Marital status with female		
Never married	322	76.8
Married	68	16.2
Cohabiting	13	3.1
Divorced/separated/widowed	16	3.8
Sexual orientation		
Homosexual	260	62.1
Bisexual	106	25.3
Heterosexual	16	3.8
Uncertain	37	8.8
Number of episodes of anal sex with males in the last week		
0	245	58.5
1–4	162	38.7
5–8	10	2.4
> 8	2	0.5
Number of male sexual partners in the last 6 months		
0	127	30.3
1	121	28.9
2–6	146	34.8
> 6	25	6.0
Condom use in the most recent anal sex with other males in the last 6 months		
Yes	241	57.5
No	51	12.2
No anal sex	127	30.3
Condom breakage, slippage or intentional removal when having sex with other males in the last 6 months		
Yes	23	5.5
No	253	60.4
Unknown	16	3.8
No anal sex	127	30.3
Use of illicit drugs		
Yes	33	7.9
No	386	92.1
Frequency of drinking alcohol in the last 3 months		
0	147	35.1
1–2 times in the last 3 months	127	30.3
1–3 times per month	91	21.7
1–4 times per week	39	9.3
> 4 times per week	15	3.6
Diagnosis of STD in the past 12 months		
Yes	22	5.3
No	397	94.7
Frequency of HIV testing		
Never	77	18.4
Tested more than 12 months ago	50	11.9
Tested once in the last 12 months	136	32.5
Tested more than once in the last 12 months	156	37.2
Ever used NPEP		
Yes	15	3.6
No	404	96.4
Locations for looking for male sexual partners		
Venues (bars, clubs, parks, and saunas	74	17.7
Internet/social media	321	76.6
Others	24	5.7
Current student		
Yes	103	24.6
No	316	75.4
Anticipated behavioral discrepancy ^b^		
0 (no discrepancy)	170	40.6
1	126	30.1
2	95	22.7
3	22	5.2
4 (the highest discrepancy)	6	1.4

***Note***. ^a^ One CYN was about 0.1486 USD. ^b^ Anticipated behavioral discrepancy was calculated as the sum of absolute values of anticipated individual change minus anticipated population change in condom use and the number of sexual partners respectively. Higher scores indicate larger discrepancy between anticipated individual and population behavior in condom use and number of sexual partners as a whole

Participants reported different perceptions about their own behaviors and other MSM’s behaviors if nPEP services are routinized. In terms of condom use, 9.8% of participants anticipated a decrease of condom use for themselves, while a third (34.2%) anticipated reduced condom use for other MSM. In terms of number of sexual partners, 16.0% of participants reported increased number of sexual partners for themselves, while more than half (54.2%) of participants anticipated increased number of sexual partners for other MSM. Correlation analyses found that the four variables were mutually and significantly correlated (see Table 2).

**Table 2:** Individual and population differences of anticipated changes on condom use and number of sexual partners among Chinese MSM if NPEP services are routinized, 2018 (*N* = 419)

	Condom use		Number of sexual partners	
	Individual	Population	Individual	Population
Increase	40.3% (169/419)	38.4% (161/419)	16.0% (67/419)	54.2% (227/419)
No change	49.9% (209/419)	27.4% (115/419)	66.8% (280/419)	30.1% (126/419)
Decrease	9.8% (41/419)	34.2% (143/419)	17.2% (72/419)	15.7% (66/419)

*Note*. Correlation analyses found that the four variables were mutually and significantly correlated: anticipated individual condom use with anticipated individual number of sexual partners (rho = 0.207, *p* < 0.001), anticipated population condom use with anticipated population number of sexual partners (rho = 0.307, *p* < 0.001), anticipated individual condom use with anticipated population condom use (rho = 0.568, *p* < 0.001), anticipated individual number of sexual partners with anticipated population number of sexual partners (rho = 0.465, *p* < 0.001), anticipated individual condom use with anticipated population number of sexual partners (rho = 0.163, *p* = 0.001), anticipated individual number of sexual partners with anticipated population condom use (rho = 0.186, *p* < 0.001)

### Measurement models of HIV knowledge and nPEP knowledge

For HIV knowledge, CFA showed that all items loaded significantly on their corresponding factor, with standardized factor loading ranging from 0.483 to 0.905 and *p* < 0.001. The goodness-of-fit of the measurement model was good (CFI = 0.970, TLI = 0.958, RMSEA = 0.043, 90% CI 0.017–0.065). For nPEP knowledge, CFA showed that all items loaded significantly on their corresponding factors, with standardized factor loading ranging from 0.731 to 0.999 and *p* < 0.001 (see Table 3). The goodness-of-fit of the measurement model was acceptable (CFI = 0.998, TLI = 0.997, RMSEA = 0.056, 90% CI 0.038–0.074).

**Table 3:** Unstandardized and standardized loading for measurement models of HIV knowledge scale and NPEP knowledge scale among Chinese MSM, 2018 (*N* = 419)

Parameter estimate	Unstandardized loading (*SE*)	Standardized loading (*SE*)
HIV Knowledge Scale confirmatory factor analysis model Fit indices: CFI = 0.970, TLI = 0.958, RMSEA = 0.043, 90% CI: 0.017–0.065		
HIV → B1	1.000	0.483 (0.080) ***
HIV → B2	1.136 (0.219) ***	0.548 (0.075) ***
HIV → B3	1.208 (0.261) ***	0.583 (0.074) ***
HIV → B4	1.321 (0.248) ***	0.638 (0.058) ***
HIV → B5	1.711 (0.329) ***	0.826 (0.090) ***
HIV → B6	1.667 (0.290) ***	0.805 (0.051) ***
HIV → B7	1.874 (0.316) ***	0.905 (0.051) ***
HIV → B8	1.366 (0.271) ***	0.659 (0.074) ***
NPEP Knowledge Scale confirmatory factor analysis model Fit indices: CFI = 0.998, TLI = 0.997, RMSEA = 0.056, 90% CI 0.038–0.074		
NPEP → A	1.000	0.875 (0.019) ***
NPEP → B	1.102 (0.025) ***	0.965 (0.013) ***
NPEP → D	1.141 (0.029) ***	0.999 (0.014) ***
NPEP → E	0.835 (0.036) ***	0.731 (0.032) ***
NPEP → F	0.988 (0.028) ***	0.865 (0.022) ***
NPEP → G	0.874 (0.035) ***	0.765 (0.030) ***
NPEP → H	1.041 (0.023) ***	0.911 (0.016) ***
NPEP → I	1.107 (0.026) ***	0.969 (0.013) ***
NPEP → K	0.905 (0.032) ***	0.793 (0.026) ***

*Note*. As suggested by the modification indices, adjustment of the confirmatory factor analysis model is not needed. Standard errors are in the parenthesis. * *p* < 0.05 ** *p* < 0.01 *** *p* < 0.001

### Structural equation model of anticipated individual behavioral changes with nPEP knowledge, HIV knowledge, and covariates among Chinese MSM

**Fig. 1:**
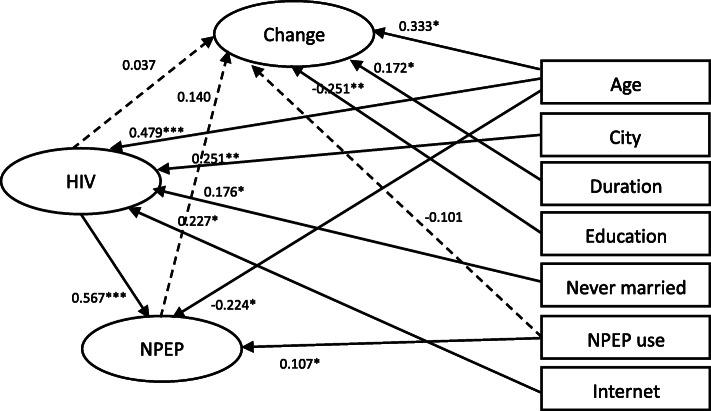
Structural equation model of anticipated individual behavioral change with HIV knowledge, NPEP knowledge and covariates among Chinese MSM (*N* = 419). Note. * *p* < 0.05, ** *p* < 0.01, *** *p* < 0.001; Fit index: RMSEA = 0.013, 90% CI 0.000–0.021, CFI = 0.995, TLI = 0.994; HIV = HIV knowledge, Change = anticipated individual behavioral change, NPEP = NPEP knowledge, Age = age, City = city of residence, Duration = duration of local residence in the current city, Education = level of education, Never married = never married, NPEP use = the use of NPEP, Internet = mainly looking for male sexual partners through the Internet. The full model estimated the relationships among anticipated individual behavioral change (the outcome), HIV knowledge, NPEP knowledge, and all covariates, but only significant paths were shown in solid lines for simplicity. Dotted lines were the primary paths of interest but not statistically significant. All path coefficients shown were standardized. The oval represents a latent construct measured by multiple items which are not shown in the diagram for simplicity

For anticipated behavioral changes on individual MSM, the structural equation model (see Fig. 1) indicated good fit indexes (RMSEA = 0.013, 90% CI 0.000–0.021, CFI = 0.995, TLI = 0.994). Anticipated individual behavioral changes were was not significantly related to the use of nPEP (b = − 0.101, *p* = 0.185), HIV knowledge (b = 0.037, *p* = 0.782), or nPEP knowledge (b = 0.140, *p* = 0.232). Instead, they were associated with age (b = 0.333, *p* < 0.05), duration of residence in the current city (b = 0.172, *p* < 0.05), and education (b = − 0.251, *p* < 0.01). Anticipated individual behavioral changes were not significantly related to the use of nPEP (b = − 0.101, *p* = 0.185), HIV knowledge (b = 0.037, *p* = 0.782), or nPEP knowledge (b = 0.140, *p* = 0.232). nPEP knowledge was associated with HIV knowledge, age, and use of PEP. HIV knowledge was associated with age, city of residence, never getting married, and mainly looking for sexual partners through the Internet.

### Structural equation model of anticipated population behavioral changes with nPEP knowledge, HIV knowledge, and covariates among Chinese MSM

**Fig. 2:**
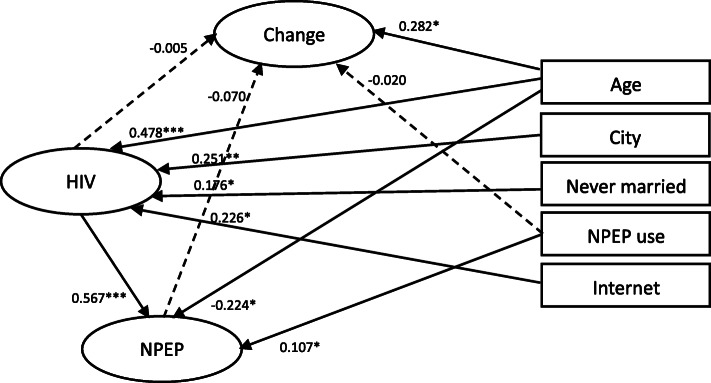
Structural equation model of anticipated population behavioral change with HIV knowledge, NPEP Knowledge and covariates among Chinese MSM (*n* = 419). Note. * *p* < 0.05, ** *p* < 0.01, *** *p* < 0.001; Fit index: RMSEA = 0.012, 90% CI 0.000–0.021, CFI = 0.996, TLI = 0.995; HIV = HIV knowledge, Change = anticipated population behavioral change, NPEP = NPEP knowledge, Age = age, City = city of residence, Never married = never married, NPEP use = the use of NPEP, Internet = mainly looking for male sexual partners through the Internet. The full model estimated the relationships among anticipated population behavioral change (the outcome), HIV knowledge, NPEP knowledge, and all covariates, but only significant paths were shown in solid lines for simplicity. Dotted lines were the primary paths of interest but not statistically significant. All path coefficients shown were standardized. The oval represents a latent construct measured by multiple items which are not shown in the diagram for simplicity

For anticipated behavioral changes on MSM population, the structural equation model (see Fig. 2) indicated good fit indexes (RMSEA = 0.012, 90% CI 0.000–0.021, CFI = 0.996, TLI = 0.995). Anticipated population behavioral changes were was not significantly related to the use of nPEP (b = 0.020, *p* = 0.730), HIV knowledge (b = − 0.005, *p* = 0.965), or nPEP knowledge (b = − 0.070, *p* = 0.472). Instead, they were only associated with age (b = 0.191, *p* < 0.05). Anticipated population behavioral changes were not significantly related to the use of nPEP (b = 0.02, *p* = 0.73), HIV knowledge (b = − 0.005, *p* = 0.965), or nPEP knowledge (b = − 0.07, *p* = 0.472). nPEP knowledge was associated with HIV knowledge, age, and use of nPEP ever. HIV knowledge was associated with age, city of residence, never getting married, and looking for sexual partners through the Internet mainly.

### Structural equation model of anticipated behavioral discrepancy with nPEP knowledge, HIV knowledge, and covariates among Chinese MSM

**Fig. 3:**
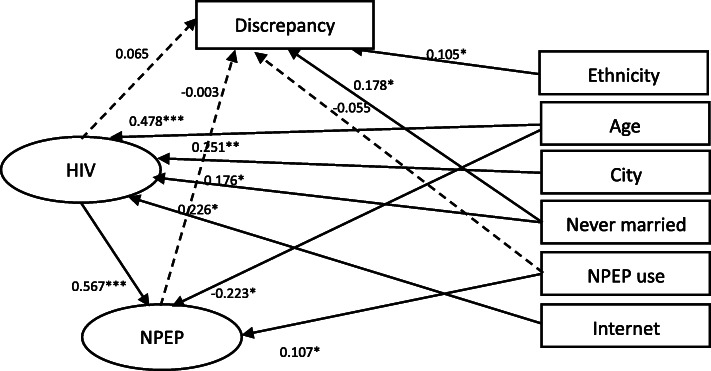
Structural Model of anticipated behavioral discrepancy with HIV knowledge, PEP knowledge, and covariates among Chinese MSM (*n* = 408). Note. * *p* < 0.05, ** *p* < 0.01, *** *p* < 0.001; Fit index: RMSEA = 0.014, 90% CI 0.000–0.023, CFI = 0.994, TLI = 0.993; HIV = HIV knowledge, Discrepancy = anticipated behavioral discrepancy, NPEP = NPEP knowledge, Ethnicity = being non-Han Chinese, Age = age, City = city of residence, Never married = never married, NPEP use = the use of NPEP, Internet = mainly looking for male sexual partners through the Internet. The full model estimated the relationships among anticipated behavioral discrepancy (the outcome), HIV knowledge, NPEP knowledge, and all covariates, but only significant paths were shown in solid lines for simplicity. Dotted lines were the primary paths of interest but not statistically significant. All path coefficients shown were standardized. The oval represents a latent construct measured by multiple items which are not shown in the diagram for simplicity

The structural equation model (see Fig. 3) indicated good fit indexes (RMSEA = 0.014, 90% CI 0.000–0.023, CFI = 0.994, TLI = 0.993). Anticipated behavioral discrepancy was associated with being non-Han Chinese (b = 0.105, *p* < 0.05), and never getting married (b = 0.178, *p* < 0.05). Anticipated behavioral discrepancy was not significantly related to the use of nPEP (b = − 0.055, *p* = 0.332), HIV knowledge (b = 0.065, *p* = 0.456), or nPEP knowledge (b = − 0.003, *p* = 0.965). nPEP knowledge was associated with HIV knowledge, age, and use of nPEP ever. HIV knowledge was associated with age, city of residence, never getting married, and mainly looking for sexual partners through the Internet.

## Discussion

To the best of our knowledge, this is the first study from China to explore anticipated behavioral change in terms of condom use and number of sexual partners from MSM’s perspective if nPEP were to be provided nationally. We found a significant proportion (50%) of participants reported their sexual behaviors may change. About 60% reported anticipated discrepancies between their personal behaviors and other men’s behaviors. nPEP use as well as HIV and nPEP knowledge were not significantly related to the anticipation of individual and population behavioral changes and discrepancy regarding condom use and number of sexual partners, which is consistent with previous studies reporting that nPEP would not lead to sexual risk behavior disinhibition [4, 13–15].

Our study has found some factors related to the anticipation of individual and population behavioral changes. Age is an important factor, since older men were more likely to state they will use more condoms and have fewer sexual partners if nPEP is available. It is reasonable to assume that as these men get older, they may have more social and sexual experiences regarding condom use and sexual partners. As HIV/AIDS prevention and intervention programs continue to focus on key groups of men who have sex with men, these men are more likely to be exposed to health promotion messages regarding consistent condom use and reducing number of sexual partners, and are therefore more likely to perceive positive (i.e., less risky) behavioral changes [37, 38]. Also, when these men have longer duration of residence in these large metropolitan cities with comparatively more complex MSM networks and more likelihood to be exposed to HIV/AIDS campaigns and messages, they might be more likely to understand the importance of using more condoms and having fewer sexual partners. Participants with higher education were more likely to state they would use less condoms and have more sexual partners if nPEP is available, indicating that these well-educated MSM might be overly optimistic about the protective effect of nPEP. These findings are different from a study conducted in the US reporting no significant differences in age or education level between those who reported condomless anal sex during nPEP and those who did not [9]. HIV/AIDS treatment optimism may be a concern among Chinese MSM, particularly those who are well-educated [13]. This is consistent with foreign studies reporting a higher incidence of sexually transmitted diseases was associated with perceiving less HIV/AIDS threat since highly active antiretroviral therapy availability [39]. A longitudinal study reported that nPEP was more often prescribed for a sexual accident with a known HIV-positive sex partner in a later period, indicating treatment optimism is a concern (41.0 versus 24.5%, *P* < 0.01) [10].

Another interesting finding is that there are differences of anticipated behavioral changes in condom use and number of sexual partners between what men anticipate for themselves and how they believe other men would respond to the availability of nPEP. These men seem to have more positive anticipations about themselves, with more negative anticipations about how their peers in the wider MSM community would react to the availability of nPEP. In other words, these men may not trust their peers, which might be about an issue of stigma related to same-sex sexuality (e.g., spoiled identity) among these marginalized and stigmatized sexual minorities [40]. This is consistent with an ethnographic study among Chinese MSM reporting men internalized a stigma that MSM are bad and dangerous and MSM communities are disappointing and hopeless [41]. Participants whose ethnicity was not Han Chinese and participants who were never married to or cohabiting with a woman were more likely to report a discrepancy between anticipated individual and population behavioral changes. Being both an ethnic minority and an MSM may make these men even more distrustful toward their MSM peers in these metropolitan cities. For MSM who never get married and never cohabit with females, they may be more comfortable with their same-sex sexuality, which is a marginalized sexuality in China. A study reported that some MSM in metropolitan areas have tried to negotiate same-sex sexuality as a normal variation of human sexuality [42]. This practice of changing social norms is difficult, as another study reported that most (70%) MSM got married to females after the age of 30 [43]. It is therefore understandable that MSM who never get married and never cohabit with females might have more negative perceptions about the MSM population.

Our study has important public health implications. The real use of nPEP was not significantly associated with anticipated behavioral changes, indicating that providing nPEP to MSM may not lead to their negative behavioral change regarding less condom use and more sexual partners. HIV and nPEP knowledge were not related to anticipated behavioral changes from the individual or population perspectives, indicating that health behavior promotion for increasing condom use and reducing number of sexual partners cannot just rely on distribution of knowledge. Most participants reported discrepancies between anticipated individual and population behavioral changes, suggesting distrust and internalized stigma among the MSM communities. It is important to be reflective about previous HIV/AIDS prevention and intervention projects, which largely focused on technical issues, such as testing tools and treatment regimens, with much less attention paid to the active and positive development of the communities from the perspective of the key populations. In this regard, when nPEP is incorporated into public health services, it needs to be implemented as a means of community development for MSM. For example, community-based MSM organizations and related stakeholders should be actively recruited to play important roles in the development and implementation of nPEP services at every step.

The current study is subject to limitations. First, the use of convenience sampling and relatively small sample size would limit the generalizability of the findings. Second, regarding the outcomes of anticipated behavioral changes, these are self-reported data and social desirability may be a concern, although this may be mitigated by the anonymous nature of the survey. MSM-related stigma may not be the only potential cause of these men’s anticipated behavioral discrepancy, which needs further examinations in future studies. Also, the concept and measurement of anticipated behavioral discrepancy should be further developed and validated. Third, stated behaviors are different from actual behaviors. However, in the context of examining nPEP as a potential national strategy for HIV prevention, the investigation of anticipated behavioral changes can provide valuable data for strategy planning and policy development. Fourth, qualitative studies exploring the whole process and in-depth experiences of using nPEP services from users’ perspective are warranted, so that researchers can develop better knowledge of using nPEP services for health behavior promotion for the key population.

## Conclusions

This study identified a high-risk subgroup of MSM who reported they would use condoms less and/or have more sexual partners when nPEP becomes available. These men were younger in age, had shorter duration of residence in the metropolitan city, and had higher education. Tailored health interventions might be needed to mitigate potential increases in sexual risk behaviors for this subgroup of MSM. Moreover, there is a clear discrepancy between anticipated individual and population behavioral changes regarding future routinization of nPEP services, suggesting the need to address the issue of distrust and internalized stigma among MSM population.

## Data Availability

The datasets used and/or analyzed during the current study are available from the corresponding author on reasonable request.

## Abbreviations

AIDS: Acquired immune deficiency syndrome
CBO: Community-based organization
CFA: Confirmatory factor analysis
CFI: Comparative fit index
CI: Confidence interval
HIV: Human immunodeficiency virus
MSM: Men who have sex with men
nPEP: Non-occupational Post-exposure Prophylaxis
CYN: China Yuan
RMSEA: Root mean square error of approximation
SD: Standardized deviation
SEM: Structural equation modeling
SPSS: Software package for statistical analysis
STI: Sexually transmitted infection
TLI: Tucker Lewis index
USD: United States dollar
